# Association of serum mannose-binding lectin, anti-phospholipase A2 receptor antibody and renal outcomes in idiopathic membranous nephropathy and atypical membranous nephropathy: a single center retrospective cohort study

**DOI:** 10.1080/0886022X.2022.2048016

**Published:** 2022-03-10

**Authors:** Yuchao Zhao, Meishun Cai, Zhenbin Jiang, Bao Dong, Yu Yan, Yina Wang, Li Zuo

**Affiliations:** Department of Nephrology, Peking University People’s Hospital, Beijing, China

**Keywords:** Idiopathic membranous nephropathy, atypical membranous nephropathy, complement, PLA2R, MBL

## Abstract

**Objectives:**

Idiopathic membranous nephropathy (iMN) is a major cause of nephrotic syndrome. Atypical membranous nephropathy (aMN) is a new type of nephropathy in China, characterized by a ‘full-house’ on immunofluorescent examination, that is IgG, IgA, IgM, C3, C1q positive, but without clinical evidence of a secondary cause. Phospholipase A2 receptor (PLA2R) was the major target antigens in iMN patients. Activation of the mannose-binding lectin (MBL) pathway plays a vital role in the development of MN. Our objective was to investigate the role of PLA2R and MBL in the pathogenesis of iMN and aMN.

**Methods:**

We conducted a retrospective observational study using propensity score matching by age, gender, and eGFR. All clinical, laboratory data, and follow-up data of the patients were collected. Serum levels of anti-PLA2R antibodies and MBL were tested.

**Results:**

Finally, 30 iMN patients and 30 aMN patients were included, and 20 healthy controls were retrospectively collected in this study. The 24 h proteinuria level was higher and serum albumin was lower in anti-PLA2R (+) patients than in anti-PLA2R (−) patients in both iMN and aMN groups. In aMN patients, MBL levels were significantly higher in anti-PLA2R (+) patients than in anti-PLA2R (−) patients (*p* = .045). The serum level of anti-PLA2R positively correlated with no-remission in both iMN and aMN groups.

**Conclusions:**

The complement lectin pathway has an association with the development of MN, especially in patients with positive anti-PLA2R antibodies. Serum MBL cannot differentiate between the two diseases. Serum MBL levels are not associated with clinical manifestations, nor with prognosis.

## 1. Introduction

Membranous nephropathy (MN) is the most common cause of nephrotic syndrome in adults and can be grouped into idiopathic membranous nephropathy (iMN) without identified causes and secondary membranous nephropathy (sMN), which is secondary to immune disease, infection, tumors, or any other cause [1]. The renal pathology of iMN is characterized by the deposition of subepithelial immune deposits that consist mainly of immunoglobulin (Ig) G and complement [2], indicating that the complement system plays a substantial role in iMN. Atypical membranous nephropathy (aMN) is a new type of membranous nephropathy characterized by cells proliferation, multi-site immune complex deposition, most of patients showed ‘full house’ in immunofluorescence, including IgA, IgG, IgM, C3, C1q positive, but no clinical evidence of a secondary cause, also called ‘lupus-like’ membranous nephropathy or ‘full-house’ membranous nephropathy [3,4]. It is controversial whether this is a new type of membranous nephropathy or an early form of secondary membranous nephropathy. From 2006 to 2015, aMN accounted for 44.39% (364 cases) of membranous nephropathy cases (820 cases) out of 3210 cases of renal puncture in our previous study [3]. The deposition of a variety of immune complexes and complement indicates that complement activation may play a role in its onset.

There are three major complement pathways include the classical pathway, mannose-binding lectin (MBL) pathway, and alternative pathway. C4d is generated in both the classical and mannose-binding lectin complement pathways. C1q is the major precursor of classical complement activation. The presence of both C4d and C1q indicates the activation of the classical pathway, but identification of C4d without C1q is more consistent with MBL pathyway; C4d and C1q are both absent in alternative complement activation [5]. The complement activation pathway in iMN remains inconclusive. IgG4, which does not activate the classical complement pathway, is the predominant immunoglobulin in iMN, whereas IgG1 and IgG3 are present in a minority of patients [6]. Deposition of C4d is detectable in essentially 100% of patients with iMN [7]; however, C1q deposits are very weak or lacking in most patients with iMN. The M-type phospholipase A2 receptor (PLA2R) has been identified as the main target antigen in the pathogenesis of iMN [8]. Some evidence suggests that IgG4 anti-PLA2R antibodies can bind MBL to activate the lectin complement pathway [9]. Based on this evidence, we hypothesized that MBL-initiated complement activation is involved in the pathogenesis of iMN. Our objective was to identify the role of MBL in the pathogenesis of iMN and aMN, and to identify the association between MBL, anti-PLA2R antibodies, and clinical outcomes in patients with MN.

## 2. Methods

### 2.1. Patients selection

This was a retrospective study. Enrolled patients were diagnosed as iMN or aMN at Peking University People’s Hospital between January 2006 to January 2015, patients were followed up until June 2020. The inclusion criteria were as follows: (1) iMN group: patients with MN characterized by immune complex deposition under the epithelium and thickening of the glomerular basement membrane; with unknown etiology; (2) aMN group: patients with MN whose renal pathology showed cell proliferation, glomerular basement membrane lesions, immune complex deposition in multiple locations of the kidney, and excluded lupus nephritis, hepatitis B virus-related glomerulonephritis, and MN secondary to drugs, toxins, and other known etiologies; (3) healthy controls: patients undergoing physical examination with normal kidney function in our hospital in 2020 matched according to their age.

Exclusion criteria: Patients with secondary MN, such as lupus nephritis, hepatitis B virus infection, medication, infections, or malignancy [10]; Patients with MN accompanied by other pathological patterns, such as diabetic nephropathy, IgA nephropathy, were also ruled out; patients withdrew during the follow-up period.

All patients were treated according to the 2012 Kidney Disease Improving Global Outcomes guidelines. Complete remission (CR) was defined as urinary protein <0.3 g/d, partial remission (PR) was defined as urinary protein 0.3–3.5 g/d and decreased >50% from the baseline value. Overall remission means CR and PR. Renal dysfunction was defined as a  ≥ 30% decline in eGFR or ESRD (end stage renal disease).

We further restricted matching by age, gender, and eGFR. The final sample included 30 iMN patients and 30 aMN patients. The primary endpoint was defined as the clinical outcomes, such as renal disfunction and overall remission.

This study was approved by the ethics committee of Peking University People’s Hospital (2017PHB141-01).

### 2.2. Clinical and laboratory data collection

The demographic, clinical, and laboratory data of enrolled patients were collected, including sex, age, blood pressure, smoking status, liver and renal function, routine urine, and urinary protein levels. Furthermore, we analyzed the serum levels of IgA, IgG, IgM, C3, and C4. Commercial enzyme-linked immunosorbent assay kits were used to detect the levels of serum MBL and anti-PLA2R antibodies in all patients with MN. All serum samples used to detect MBL and anti-PLA2R antibodies were obtained from the Biological Sample Bank of the People’s Hospital. Anti-PLA2R antibody positivity was defined as a level greater than 20 U/mL. Renal puncture tissue was examined using optical microscopy (HE, Masson, PASM staining), electron microscopy, and immunofluorescence. Immunofluorescence analysis included the assessment of IgA, IgG, IgM, C1q, C3, IgG1, IgG2, IgG3, and IgG4 expression.

### 2.3. Statistical analysis

SPSS 22.0 software was used for statistical analysis. Variables with a normal distribution are expressed as mean ± standard deviation and were compared using t-tests, and data with a non-normal distribution are presented as the median and quartile and were compared using a nonparametric test. Categorical variables were compared using the χ^2^ test. Correlations were analyzed using Pearson’s correlation test (between two normally distributed variables) or Spearman’s correlation test (between two non-normally distributed variables). Two groups of patients were matched with 1:1 propensity score matching (PSM) method. Patient renal outcomes were analyzed using the Kaplan–Meier method. Logistic regression analysis was used to examine risk factors for no-remission in the patients with iMN and aMN.Statistical significance was set at *p* < .05.

## 3. Results

### 3.1. Patients’ clinical and pathological features

A total of 145 iMN patients and 153 iMN met the criteria, 4 patients were excluded for lacking follow up data Before PSM (Table 1), patients with iMN were significantly older than patients with aMN (55.2 ± 11.7 vs. 46.5 ± 13.4 years old, *p* = .021), and eGFR of iMN patients is lower than aMN patients. After 1:1 PSM, 60 patients (30 patients in each group) were selected for further statistical analysis, and no significant difference was then identified between the two groups in the age and eGFR (*p* > .05). Mean serum albumin concentration was 26.3 g/L and 24.9 g/L in the iMN and aMN groups, respectively (*p* = .5). The serum anti-PLA2R antibody positivity rate was 56.67% in the iMN group and 70.00% in the aMN group, which was not significantly different (*p* = .284). The median serum MBL levels were 1138.50 ng/mL, 799.00 ng/mL, and 681.00 ng/mL in the iMN, aMN, and healthy control groups, respectively. The serum levels of MBL in patients with iMN were higher than aMN patients (*p* = .045) and healthy controls (*p* = .021). The general information and baseline clinical characteristics of patients are shown in Table 2.

**Table 1.** Demographic and clinical characteristics of enrolled patients before and after PSM matching.

	Before PSM matching			After PSM matching		
Characteristics	iMN group (*n* = 143)	aMN group (*n* = 151)	*p*	iMN group (*n* = 30)	aMN group (*n* = 30)	*p*
Age (years)	55.2 ± 11.7	46.5±13.4	.021	56.9 ± 10.4	55.9 ± 11.1	.729
Gender (male, *n*%)	77 (53.8%)	86 (56.9%)	.657	53.3	46.6	.787
eGFR (ml/min/1.73m2)	90.62 ± 20.02	97.82 ± 23.03	.006	95.21 ± 13.42	95.94 ± 14.47	.830

PSM, propensity score matching; iMN, idiopathic membranous nephropathy; aMN, atypical membranous nephropathy

**Table 2.** Demographic and clinical characteristics of enrolled patients. iMN: idiopathic membranous nephropathy; aMN: atypical membranous nephropathy;PLA2R: phospholipase A2 receptor; MBL: mannose-binding lectin;

	iMN group (*n* = 30)	aMN group (*n* = 30)	*p*
Smoking rate (*n*, (%)	9 (30%)	11 (36.7%)	.584
Albumin (g/L)	26.32 ± 8.38	24.91 ± 7.76	.500
Proteinuria (g/24 h)	3.57 (1.71,6.64)	5.74 (3.86,5.88)	.060
Serum creatinine (μmol/L)	70.23 ± 14.448	69.33 ± 15.359	.882
Uric acid (mmol/L)	394.03 ± 120.913	368.23 ± 115.103	.401
Triglyceride (mmol/L)	2.69 ± 1.04	2.89 ± 2.34	.472
Cholesterol (mmol/L)	7.21 ± 2.242	7.52 ± 2.68	.521
Serum IgA (g/l)	2.01 ± 0.80	2.28 ± 1.25	.324
Serum IgG (g/l)	5.8 (3.77, 7.20)	5.8 (3.92, 9.02)	.048
Serum IgM (g/l)	0.90 ± 0.429	1.07 ± 0.54	.182
Serum C3 (g/l)	1.09 ± 0.2	1.11 ± 0.24	.691
Serum C4 (g/l)	0.29 (0.24, 0.32)	0.24 (0.18, 0.28)	.971
Anti-PLA2R antibody (RU/ml)	31.00 (1.00, 74.75)	39.50 (2.50,96.00)	.550
Anti-PLA2R antibody positive (*n*, %)	17 (56.67)	21 (70.00)	.284
MBL (ng/ml)	1138.50 (478.62, 1538.37)	799.00 (246.75, 1247.62)	**.045**

### 3.2. Immunofluorescence tests of renal biopsies in patients with iMN and aMN

As shown in Table 3, IgG was detectable in 100% of patients with iMN, IgG4 was the predominant immunoglobulin in iMN (80%), whereas IgG1, IgG2, and IgG3 were present in 56.7%, 10%, and 3.3% of patients with iMN, respectively. Most renal immunofluorescence tests of patients with aMN were characterized by the ‘full-house’. There was a significant difference in the positive rates of IgA, IgM, C1q, and IgG2 between patients with iMN and aMN. In the aMN group, the highest positive rate was observed for IgG1 (76.7%).

**Table 3.** Immunofluorescence tests of renal biopsies in patients with iMN and aMN.

	iMN group (*n* = 30)	aMN group (*n* = 30)	*p*
IgA [positive, *n* (%)]	2 (6.7%)	20 (66.7%)	**<.0001**
IgG [positive, *n* (%)]	30 (100%)	29 (96.7%)	.313
IgM [positive, *n* (%)]	13 (43.3%)	24 (80%)	**.003**
C1q [positive, *n* (%)]	2 (6.7%)	24 (80%)	**<.0001**
C3 [positive, *n* (%)]	25 (83.3%)	29 (96.7%)	.085
IgG1 [positive, *n* (%)]	17 (56.7%)	23 (76.7%)	.1
IgG2 [positive, *n* (%)]	3 (10%)	19 (63.3%)	**<.0001**
IgG3 [positive, *n* (%)]	1 (3.3%)	2 (6.7%)	1.0
IgG4 [positive, *n* (%)]	24 (80%)	20 (66.7%)	.39

### 3.3. Differences between anti-PLA2R-positive and anti-PLA2R-negative patients in iMN patients and aMN patients

The 24-h proteinuria level was significantly higher and serum albumin was lower in anti-PLA2R-positive groups than in anti-PLA2R-negative groups in both iMN and aMN patients. There was no significant difference in serum MBL level between anti-PLA2R-positive and -negative groups in patients with iMN, but in patients with aMN, serum MBL level was significantly higher in anti-PLA2R(+) groups than in anti-PLA2R(−) groups (*p* = .04) (Table 4). There was no association between serum MBL and 24-h proteinuria in either iMN patients or aMN patients (*p* all > .05).

**Table 4.** Differences between anti-PLA2R-positive and anti-PLA2R-negative patients in iMN patients and aMN patients.

	iMN (*N* = 30)			aMN (*N* = 30)		
	PLA2R-Ab (+) *N* = 17	PLA2R-Ab (−) *n* = 13	*p*	PLA2R-Ab (+) *N* = 21	PLA2R-Ab (−) *N* = 9	*p*
Proteinuria (g/24 h)	5.83 (1.61,10.84)	3.82 (1.68,4.61)	**.017**	6.18 (3.15,8.60)	5.53 (4.16,9.31)	**.024**
Serum urea, mmol/L	6.00 ± 1.65	5.0 ± 1.13	.065	5.25 ± 1.45	5.33 ± 2.20	.922
Serum creatinine, umol/L	71.94 ± 15.22	68.01 ± 13.62	.462	69.61 ± 15.81	68.66 ± 15.13	.878
Serum albumin, g/L	23.92 ± 7.35	29.43 ± 8.89	**.048**	22.74 ± 8.86	25.84 ± 7.26	**.037**
eGFR, mL/min/1.73 m2	94.61 ± 16.73	95.97 ± 7.97	.77	98.36 ± 13.89	90.27 ± 14.98	.188
Serum MBL, ng/mL	1360 (384, 1857)	1059 (556, 1475)	.477	1110 (493, 1512)	654 (226, 1047)	**.045**
Serum IgA	1.88 ± 0.78	2.16 ± 0.80	.345	2.45 ± 1.31	1.87 ± 1.01	.206
Serum IgG	6 (4.51, 10.85)	7.51 (6.85, 11.42)	.103	6.30 (4.41, 9.15)	5.40 (3.11, 9.25)	.449
Serum IgM	0.88 ± 0.41	0.92 ± 0.46	.769	1.05 ± 0.56	1.10 ± 0.65	.840
Serum C3	1.11 ± 0.20	1.05 ± 0.19	.468	1.10 ± 0.22	1.11 ± 0.29	.931
Serum C4	0.29 (0.25, 0.35)	0.27 (0.22, 0.32)	.263	0.30 (0.25, 0.33)	0.27 (0.19, 0.36)	.929

iMN, idiopathic membranous nephropathy; aMN, atypical membranous nephropathy; PLA2R-Ab, anti-P LA2R antibodies ; MBL, mannose-binding lectin.

### 3.4. Treatment responses and renal outcomes in patients with iMN and aMN

All 60 patients received treatment with angiotensin-converting enzyme inhibitors or angiotensin receptor blockers. Fourteen (46.6%) patients were administered glucocorticoids and cyclophosphamide, and 2 (6.6%) patients were administered glucocorticoids and calcineurin inhibitors in the iMN group. In the aMN group, 17 patients (56.6%) received glucocorticoids and cyclophosphamide, and 5(16.7%) received glucocorticoids and calcineurin inhibitors.

### 3.5. Effect of serum MBL and anti-PLA2R antibody levels on treatment responses and renal outcomes

**Figure 1.**
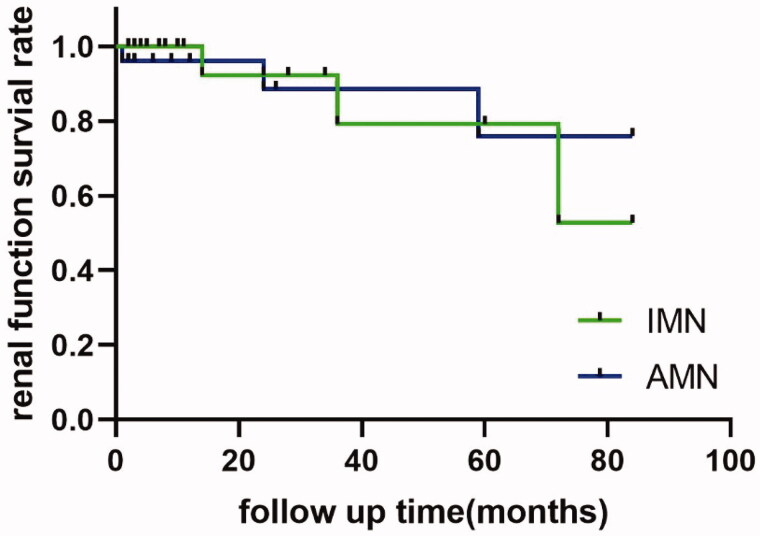
Renal functional survival rate. Survival curves of iMN and aMN groups. iMN: idiopathic membranous nephropathy; aMN: atypical membranous nephropathy;

During follow-up, 22 (73.3%) patients achieved overall remission in the iMN group, and 17 (56.7%) patients achieved overall remission in the aMN group. Two (6.7%) patients had kidney dysfunction in the iMN group; and 3(10%) patients had kidney dysfunction in the aMN group. Kaplan–Meier analysis showed no difference in renal dysfunction rates between the iMN and aMN groups (χ^2^ = 0.034, *p* = .853) (Figure 1).

The influence factors of no remission were determined by logistic regression (Table 5). After analysis, PLA2R(+) was independent factors for no remission in both iMN groups and aMN groups. but serum MBL was not independent factors for renal disfunction in iMN and aMN patients.

**Table 5.** Logistic regression analysis of the risk factors for no-remission in the patients with iMN and aMN.

	iMN group		aMN group	
Parameters	OR (95% CI)	*p*	OR (95% CI)	*p*
Anti-PLA2R antibody positivity	1.184 (1.265,7.833)	**.039**	1.114 (1.201,6.943)	**.042**
Proteinuria (g/24 h)	1.167 (0.887,1.528)	.273	1.124 (0.874,1.501)	.317
MBL	1.004 (0.992,1.016)	.491	1.017 (0.952,1.034)	.572

iMN, idiopathic membranous nephropathy; aMN, atypical membranous nephropathy; P LA2R, phospholipase A2 receptor; MBL, mannose-binding lectin.

## 4. Discussion

According to our results, serum MBL level was significantly higher in anti-PLA2R(+) groups than in anti-PLA2R(−) groups in patients with atypical membranous nephropathy, this phenomenon is not observed in patients with idiopathic membranous nephropathy. There was no association between serum MBL and renal outcomes either in iMN groups or aMN groups.

MN can be divided into idiopathic membranous nephropathy (iMN) without a definite etiology and secondary membranous nephropathy (sMN). A newly identified form of MN that is characterized by cell proliferation and a ‘full-house’ in immunofluorescence has been discovered in China, which is similar to sMN but without confirmation of the basic etiology [3,11]. Some scholars named it as atypical membranous nephropathy(aMN), but others thought it as lupus-like MN or ‘full house’ MN, no final conclusion had yet been reached on this matter. In the previous study of aMN and iMN in our center [10], The average age of patients with iMN was significantly higher than that of patients with aMN. After matching by age, gender, and eGFR, there were no significant differences in clinical manifestation; or serum IgA, IgM, C3 levels, and renal outcomes between patients with aMN and those with iMN in this study. This result is similar to that of a previous study [10,12] .

The renal pathology of iMN is characterized by the deposition of predominant IgG4 with low amounts of IgG1 and IgG3. IgG4 does not activate the classical complement pathway. C4d is detectable in essentially 100% of patients with iMN [7] which was absent in alternative complement activation [5]. These observations suggest that the MBL-initiated complement pathway may be the predominant complement activation in IMN. Mathern et al. [13] found that MBL and IgG were important components in subepithelial deposits in iMN. In a study by Lhotta et al., the glomerular MBL deposition rate was 66.7% in patients with iMN [14]. In Zhang’s study [9], glomerular MBL deposition was detected in 79.1% of patients with biopsy-proven iMN, suggesting that MBL-initiated complement pathway activation is a pathogenic factor in iMN; *in vitro* [15], Haddad G et al. showed that anti-PLA2R1 IgG4 autoantibodies were able to activate the lectin complement pathway and induce sublethal injury of PLA2R1-expressing podocytes. However, MBL activation is not the only way to develop iMN because iMN can develop in patients with complete MBL deficiency [16]. Predominant IgG4 deposits and very small IgG1 and C1q deposits in these patients indicate the role of the alternative pathway of complement activation in iMN pathogenesis. Atypical membranous nephropathy (aMN) characterized by cells proliferation, multi-site immune complex deposition, ‘full house’ in immunofluorescence, including IgA, IgG, IgM, C3, C1q positive, which implies that the pathophysiological process involves complex complement system activation.

To the best of our knowledge, no studies have summarized the relationship between serum MBL levels and aMN. In our study, serum MBL was detected in all 30 patients with iMN and 30 patients with aMN, as well as in 20 healthy controls. Serum MBL was significantly higher in patients with iMN than in those with aMN and healthy individuals respectively. There was no difference in MBL levels between anti-PLA2R-positive and-negative groups among patients with iMN (*p* = .408), which is similar to Zhang’s et al. [9] finding. However, in patients with aMN, serum MBL was higher in the anti-PLA2R-positive group than in the anti-PLA2R-negative group.

A lack of MBL can reduce the clearance of the autoantigen, thus favoring the development of autoimmunity, leading to a poor response to treatment [14]. Low serum MBL levels in patients with rheumatoid arthritis predict poor prognosis according to study by Saevarsdottir et al. [17] Guo et al. [18] found that MBL deficiency and MBL excess may have deleterious effects on IgA glomerulonephritis progression. They measured serum MBL levels in 749 patients with IgA glomerulonephritis and 489 healthy controls and found that the symptoms of hematuria and infection were significantly higher in patients with low MBL levels (<100 ng/mL) than in those with sufficient (100-3540 ng/mL) MBL levels. Patients with high MBL levels (>3540 ng/mL) had more severe proteinuria and a higher proportion of crescents. Zhang et al. [19] measured the circulating complement components in 134 patients with iMN and found that serum MBL levels positively correlated with 24 h urinary protein in anti-PLA2R antibody-positive patients, but not in anti-PLA2R antibody-negative patients. MBL levels had no predictive value for treatment responses or renal outcomes in patients with iMN. They thought that complement may be activated through the lectin pathway in anti-PLA2R antibody-positive patients and through alternative pathways in anti-PLA2R antibody-negative patients. Our results showed that there was no difference in serum MBL level between anti-PLA2R(+) and anti-PLA2R(−)groups in patients with iMN; however, serum MBL levels were significantly higher in anti-PLA2R-positive groups than in negative groups in patients with aMN. There were no correlations between serum MBL levels and renal outcomes in iMN and aMN groups. Our study cannot prove causation between MBL and aMN and iMN yet, it at least shows that MBL cannot distinguish the two kinds of disease, future studies with larger samples are needed to explore the pathogenesis of aMN and iMN.

This study had several limitations. Firstly, this study was retrospective and conducted at a single center. Secondly, the study sample was relatively small, which may affect the stability of statistical results. Finally, we regret that we were not able to detect MBL, PLA2R, C5b–9, and C4d in renal tissue samples retrospectively yet, due to lack of funding, and the workload of re-pathological sections.

## 5. Conclusion

In conclusion, our study investigated the association between serum MBL levels and renal outcomes in patients with iMN and aMN in China. MBL pathway of complement activation plays an important role in the pathogenesis of iMN. Patients with aMN are characterized by a ‘full-house’ on immunofluorescence, hinting at more complex complement activation in the aMN pathological process. Serum MBL cannot differentiate between the two diseases. We did not find any association between serum MBL levels and clinical manifestations and renal outcomes. Further studies are still needed to investigate the pathogenesis of aMN.
